# Evaluation of oxygenation indices incorporating SpO₂ and PEEP for assessing ARDS severity: Evidence from the MIMIC-IV and eICU collaborative research database v2.0 databases

**DOI:** 10.1371/journal.pone.0341004

**Published:** 2026-02-05

**Authors:** Zekun Wei, Cunyang Li, Zhiyun Liu, Bolin Wang, Can Wang, Yang Liu, Tejin Ba, Li Kong, Feihu Zhang

**Affiliations:** 1 The First Clinical Medical College, Shandong University of Traditional Chinese Medicine, Jinan, China; 2 Department of Emergency Center, Shandong University of Traditional Chinese Medicine Affiliated Hospital, Jinan, China; 3 Department of Emergency and Critical Care Medicine, International Mongolian Medical Hospital of Inner Mongolia Autonomous Region, Hohhot, China; Bilawal Medical College, Liaquat University of Medical and Health Sciences, PAKISTAN

## Abstract

**Background:**

The in-hospital mortality of acute respiratory distress syndrome can reach 35–45%, with patients requiring a more convenient and accurate way to assess the disease condition, which can change even more rapidly in patients undergoing mechanical ventilation in intensive care units.

**Methods:**

Eligible patients in MIMIC-IV v3.0and eICU Collaborative Research Database v2.0were screened by the Berlin definition to examine the comparison of the diagnostic abilities of SpO_2_*10/FiO_2_*PEEP (S/F*P), PaO_2_*10/FiO_2_*PEEP (P/F*P), and SpO_2_/ FiO_2_ (S/F), with nine types of machine learning performed on S/F*P for 10 cross-validations, validating the diagnostic ability of the models for ARDS patients.

**Results:**

ROC_AUC = 0.700(95 CI:0.624 ~ 0.777) for S/F, ROC_AUC = 0.720(95 CI:0.668 ~ 0.772) for P/F*P, and ROC_AUC = 0.761(95 CI:0.693 ~ 0.830) for S/F*P showed that S/F had a better fit in diagnosing ARDS with slightly inferior efficacy to P/F*P, had superior diagnostic efficacy after incorporating peep into S/F, and S/F*P showed good diagnostic efficacy in 9 machine learning and 10 cross-validations. In terms of predicting the prognosis of patients, the ability of S/F*P is not as good as S/F, but the grading of S/F*P has a more positive significance for the evaluation of the prognosis of patients.

**Conclusion:**

S/F*P provides a more convenient judgment for mechanically ventilated patients, avoiding the phenomenon of clinical diagnosis of PEEP and oxygenation index separation as much as possible, minimizing invasive operation of patients and improving the selection of ARDS treatment modalities. Therefore, S/F*P provides a reference for the early treatment of ARDS in the clinic to improve the resource allocation in the ICU and reduce the mortality of patients, Given all patients had ARDS diagnosis, this study evaluated relative diagnostic performance among indices rather than disease vs. non-disease discrimination.

## Introduction

Acute respiratory distress syndrome (ARDS) is a life-threatening respiratory failure characterized by severe hypoxemia due to inflammatory pulmonary edema [1], which means ARDS is an acute diffuse lung injury associated with susceptible risk factors and is characterized by inflammation-induced pulmonary vascular permeability increase and alveolar collapse. The hallmarks are hypoxemia and bilateral x-ray infiltrates, increased right-to-left pulmonary venous mixing, increased physiologic dead space, and decreased respiratory compliance. Diffuse alveolar damage (i.e., pulmonary edema, inflammation, vitreous membranes, and alveolar hemorrhage) is the typical morphologic manifestation [2].

The 2012 Berlin definition categorizes hypoxia into low (200 mmHg < PaO_2_/ FiO_2_ ≤ 300 mmHg), moderate (100 mmHg < PaO_2_/ FiO_2_ ≤ 200 mmHg), and severe (PaO_2_/ FiO_2_ ≤ 100 mmHg) according to the degree of hypoxia [3]. A new global definition of acute respiratory distress syndrome in 2023 presents the use of SpO_2_ as a diagnosis of ARDS as a complement to the Berlin definition, which means ARDS can be diagnosed when SpO_2_ is ≤ 97% and SpO_2_/ FiO_2_ ≤ 315, 235 < SpO_2_/ FiO_2_ ≤ 315 was mild, 145 < SpO_2_/ FiO_2_ ≤ 235 was moderate, and SpO_2_/ FiO_2_ ≤ 148 was severe [4]. ARDS in-hospital mortality can be as high as 35–45%, with even a 31% mortality rate for patients having a rapid relief of ARDS symptoms. It has been observed that a mortality rate of 27% for mild ARDS, 32% for moderate ARDS, and 45% for severe ARDS [5].

The Berlin definition showed that a PEEP ≥5 cmH_2_O is the basic requirement for ARDS diagnosis, but PEEP can affect PaO_2_. Therefore, lower PEEP settings can result in more patients being labeled as having severe ARDS, with patients with higher positive pressure ventilation settings being defined as having mild or even no ARDS. This separate definition of PEEP and PaO_2_ severity leads to inaccurate predictions and uncertainty about when to implement specific therapeutic interventions [6]. The Berlin definition helps to identify severe ARDS but may be less meaningful in differentiating between mild and moderate disease [7].

In intensive care units, mechanical ventilation is essential to correct hypoxemia due to ARDS, with PEEP as an important parameter of mechanical ventilation [8]. However, there is still no PEEP for optimal treatment because of undetermined PEEP standards, which means the value of PEEP is necessary to be adjusted in time for different therapeutic needs. For example, a higher PEEP will be used to improve the patient’s condition in the case of low tidal volume, which depends more on the judgment of clinicians without a standardized PEEP adjustment strategy [9–12]. However, an inappropriate PEEP strategy can lead to further lung injury in patients [13].

Therefore, it is important to find a timely and convenient indicator to observe the occurrence of ARDS for the early prevention or treatment of ARDS in patients, which is important for the prognosis of patients. Ratios of ventilator-related parameters like SpO_2_*10/ FiO_2_ (S/F), SpO_2_*10/FiO_2_*PEEP (S/F*P), and PaO_2_*10/ FiO_2_*PEEP(P/F*P) have been studied for the prediction of ARDS incidence [6 14].

In the study, a machine learning approach was used to examine the diagnostic and prognostic value of S/F*P in mechanically ventilated patients with ARDS and compare it with several established formulas to establish convenient and validated assessment indicators for evaluating patients with ARDS undergoing mechanical ventilation.

## Methods

### Study design and patient selection

**Fig 1:**
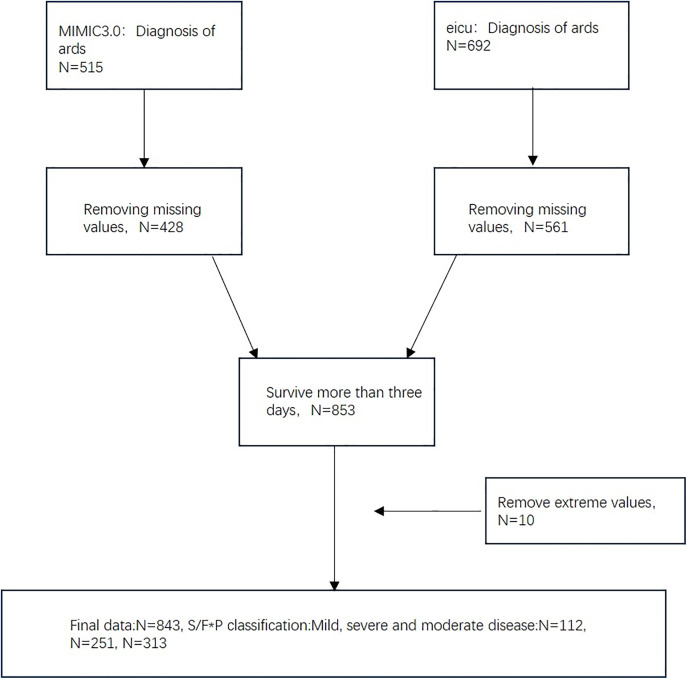
Technology Roadmap.

Patients with a confirmed diagnosis of ARDS were identified from the MIMIC-IV v3.0 and eICU Collaborative Research Databasev2.0, two publicly available, de-identified critical care datasets hosted on PhysioNet. For each patient, the variables SpO₂, FiO₂, PEEP, and PaO₂ were extracted within three predefined time intervals following ICU admission: 0–24 hours, 48–72 hours, and 72–168 hours. Within each interval, the worst recorded value (i.e., the most severe oxygenation impairment) was selected for analysis to calculate oxygenation indices, including S/F, S/F*P, P/F, and P/F*P. After excluding patients with missing data or outliers, 428 patients from the MIMIC-IV dataset and 561 patients from the eICU Collaborative Research Database v2.0 dataset were included in the final analysis (initially N = 515 and N = 692, respectively).Patients with a confirmed diagnosis of ARDS were extracted from the MIMIC-IV and eICU Collaborative Research Database v2.0 according to the Berlin definition. Because all enrolled subjects already met the diagnostic criteria for ARDS, the purpose of this analysis was not to distinguish ARDS from non-ARDS cases, but rather to compare the relative diagnostic performance among different oxygenation indices (S/F, S/F*P, and P/F*P) in evaluating ARDS severity. This approach allows internal comparison of indices within a clinically homogeneous ARDS population and reflects their practical value for classification and monitoring. To further explore the ability to predict the prognosis of patients and the ability to diagnose grading, the data of patients who survived ≥3 days (N = 853) was selected to build the formula using the last value of 3 and 7 days. Finally, the demarcation of S/P*F for different grades of ARDS was delineated based on the formula results on day 7 corresponding to the Berlin definition, where 0 is for patients without a diagnosis of ARDS on day 7 (N = 167), 1 is for patients with mild disease (N = 112), 2 is for patients with moderate disease (N = 251), and 3 is for patients with severe disease (N = 313).(Fig 1)

### MIMIC-IV v3.0 database

The publicly available MIMIC-IV v3.0 database [15–17] containing information on patient hospitalizations from 2008–2022 was used in the study, which is an important database in the field of intensive care units.

### eICU collaborative research database v2.0 database

The eICU Collaborative Research Database v2.0 is a multicenter database containing de-identified health data related to more than 200,000 ICU admissions in the United States between 2014 and 2015. The database includes vital sign measures, care plan documentation, disease severity measures, diagnostic information, and treatment information [16,18,19].

### Experimental methods

Patients with ARDS were identified by the Berlin definition; we compared diagnoses derived from S/F, S/F*P, and the traditional Berlin criteria to assess diagnostic performance across S/F, S/F*P, and P/F*P. We then visualized and analyzed predictive performance for ARDS diagnosis. For prognosis, the optimal decision threshold was set by the Youden index computed on the training dat.

Nine machine-learning classifiers—Decision Tree (DT), Random Forest (RF), XGBoost, LightGBM, Elastic Net (ENet), Support Vector Machine (SVM), Multi-Layer Perceptron (MLP), K-Nearest Neighbors (KNN), and Logistic Regression—were trained on S/F*P and S/F. Data were split with stratification (70% training, 30% testing). Hyperparameters were tuned with 10-fold cross-validation on the training set and models were then refit on the full training data and evaluated on the held-out test set; model validation used accuracy, ROC-AUC, and PR-AUC, and probability calibration curves were drawn using binning and windowed methods [19–21].

### Result evaluation

For the ROC curve, it is excellent with an AUC ≥ 0.9, it is “good” with an AUC between 0.8 and 0.9, it is “fair” with an AUC between 0.7 and 0.8, it is “poor” with an AUC between 0.6 and 0.7, and it is “very poor” with an AUC below 0.6. SHAP was used for further visual evaluation of the model results

## Results

### Patient information

Among 843 participants, 323 (38.3%) were in the Death group, while 520 (61.7%) were in the Live group. Participants in the Death group were older (mean age 62.8 ± 15.3 years vs 55.2 ± 15.6 years; p < 0.001) and had higher SOFA scores (mean 9.4 ± 3.9 vs 7.6 ± 3.1; p < 0.001). The Death group had lower SpO2 on day 1 (mean 93.1 ± 7.1 vs 94.1 ± 5.7; p = 0.024). No statistically significant differences were observed in PEEP day 1 (p = 0.321), FiO2 day 1 (p = 0.051), PaO2 day 1 (p = 0.160), tidal volume (p = 0.919), hemoglobin (p = 0.934), gender (p = 0.273), or sepsis prevalence (p = 0.972) between the groups.(Table 1)

**Table 1:** Patient information.

Variables	Total (n = 843)	Live (n = 520)	Death (n = 323)	*P*
Age	58.078 ± 15.895	55.172 ± 15.573	62.757 ± 15.302	**<.001**
SOFA	8.284 ± 3.535	7.587 ± 3.130	9.406 ± 3.853	**<.001**
PEEP day1	10.165 ± 4.536	10.042 ± 4.457	10.361 ± 4.661	0.321
SpO2 day1	93.740 ± 6.287	94.146 ± 5.671	93.085 ± 7.128	**0.024**
FiO2 day1	80.206 ± 23.800	78.960 ± 24.147	82.214 ± 23.127	0.051
PaO2 day1	115.403 ± 80.499	118.473 ± 85.781	110.461 ± 71.027	0.160
Tidal Volume	432.933 ± 91.827	433.186 ± 90.763	432.524 ± 93.655	0.919
Hb	9.700 ± 3.410	9.707 ± 3.497	9.688 ± 3.269	0.934
Gender, n(%)				0.273
F	354 (41.993)	226 (43.462)	128 (39.628)	
M	489 (58.007)	294 (56.538)	195 (60.372)	
Sepsis, n(%)				0.972
N	178 (21.115)	110 (21.154)	68 (21.053)	
Y	665 (78.885)	410 (78.846)	255 (78.947)	

### SP/F has a better diagnostic ability

**Fig 2:**
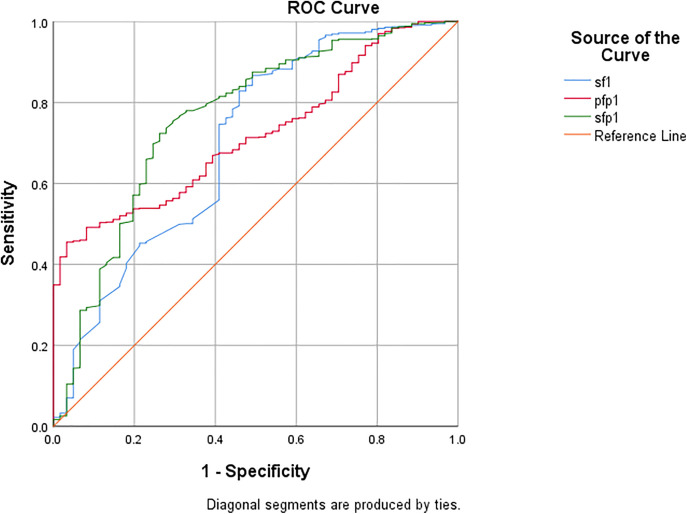
AUC curves for the diagnosis of ARDS. Sfp1:SpO2 * 10/FiO2 * PEEP (S/F*P), pfp1:PaO2 * 10/FiO2 * PEEP (P/F*P),sf1: SpO2 * 10/FiO2 (S/F).

Fig 2 and Table 2 show that ROC_AUC = 0.700(95 CI:0.624 ~ 0.777) for S/F, ROC_AUC = 0.720(95 CI:0.668 ~ 0.772) for P/F*P, and ROC_AUC = 0.761(95 CI:0.693 ~ 0.830) for S/F*P.

**Table 2:** AUC curves for the diagnosis of ARDS. SpO2 * 10/FiO2 * PEEP (S/F*P), PaO2 * 10/FiO2 * PEEP (P/F*P), SpO2 * 10/FiO2 (S/F).

Area Under the Curve				
Area	Std. Error^a^	Asymptotic Sig.^b^	Asymptotic 95% Confidence Interval	
			Lower Bound	Upper Bound
0.700	0.039	<0.001<0.001	0.624	0.777
0.720	0.027	<0.001<0.001	0.668	0.772
0.761	0.035	<0.001	0.693	0.830

### S/F has a better predictive ability of prognosis than S/F*P

**Fig 3:**
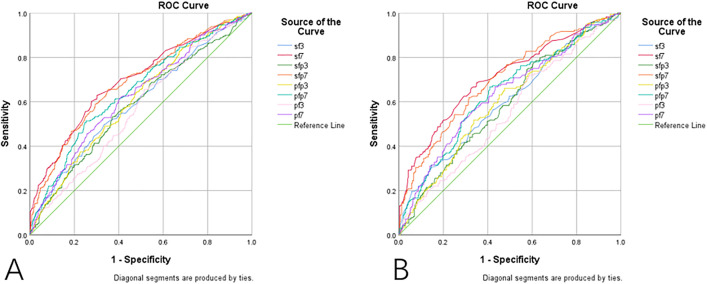
Predictive power of A.sf: S/F, sfp: S/F*P, pfp: P/F*P, and pf: P/F for prognosis in all patients B. Predictive power of sf: S/F, sfp: S/F*P, pfp: P/F*P, and pf: F/P for prognosis in patients with PEEP≥8.

In order to verify the predictive ability of S/F*P for prognosis, ROC curves were plotted for all patients and patients with PEEP ≥ 8, respectively (Fig 3, Tables 3 and 4), ultimately yielding S/F (S/F) of 0.598 and 0.603, S/F*P (S/FS/F*P) of 0.585 and 0.589, P/F*P (P/FP/F*P) of 0.608 and 0.600, and P/F (P/F) of 0.567 and 0.533 at day 3, as well as S/F (S/F) of 0.696 and 0.710, S/F*P (S/FS/F*P) of 0.686 and 0.698, P/F*P (P/FP/F*P) of 0.650 and 0.639, and P/F (P/F) of 0.626 and 0.633 at day 7.Although P/F showed the highest AUC among the indices, the overall discriminatory performance of all models remained modest (AUC < 0.7), indicating limited prognostic accuracy. Nevertheless, these indices may still provide preliminary guidance for early risk stratification in ARDS management.

**Table 3:** Predictive power of S/F, S/F*P, P/F*P, and P/F for the prognosis of all patients.

Area Under the Curve					
Test Result Variable(s)	Area	Std. Errora	Asymptotic Sig.b	Asymptotic 95% Confidence Interval	
				Lower Bound	Upper Bound
S/F3	0.603	0.027	<0.001	0.549	0.656
S/F7	0.710	0.025	<0.001	0.661	0.760
S/FS/F*P3	0.589	0.027	0.001	0.536	0.641
S/FS/F*P7	0.698	0.025	<0.001	0.649	0.747
P/FP/F*P3	0.600	0.026	<0.001	0.548	0.652
P/FP/F*P7	0.639	0.026	<0.001	0.587	0.690
P/F3	0.553	0.027	0.052	0.5	0.606
P/F7	0.633	0.027	<0.001	0.581	0.686

**Table 4:** Predictive power of S/F, S/F*P, P/F*P, and P/F for prognosis in patients with PEEP>8.

Area Under the Curve					
Test Result Variable(s)	Area	Std. Errora	Asymptotic Sig.b	Asymptotic 95% Confidence Interval	
				Lower Bound	Upper Bound
S/F3	0.603	0.027	<0.001	0.549	0.656
S/F7	0.710	0.025	<0.001	0.661	0.760
S/FS/F*P3	0.589	0.027	<0.001	0.536	0.641
S/FS/F*P7	0.698	0.025	<0.001	0.649	0.747
P/FP/F*P3	0.60	0.026	<0.001	0.548	0.652
P/FP/F*P7	0.639	0.026	<0.001	0.587	0.690
P/F3	0.553	0.027	0.052	0.5	0.606
P/F7	0.633	0.027	<0.001	0.581	0.686

### Machine Learning for S/F and S/F*P

**Fig 4:**
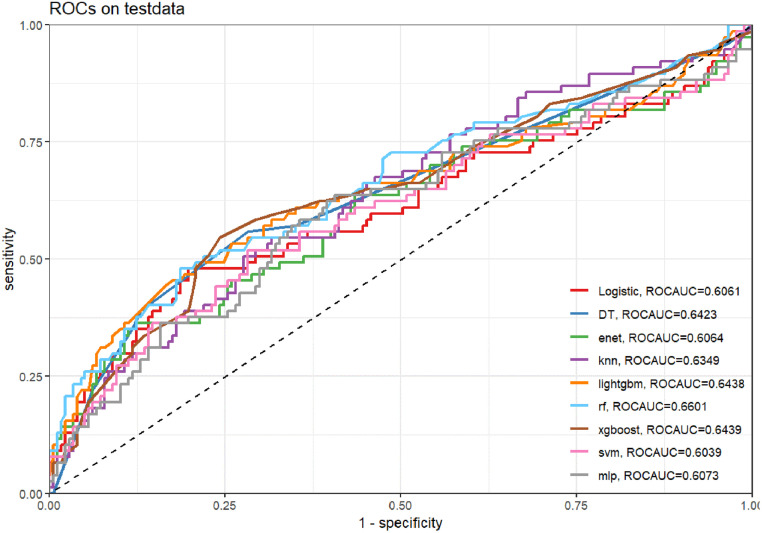
ROC_AUC for the nine machine learning models.

Detailed indicators for different models in the test set were derived through nine kinds of machine learning (Fig 4). Fig shows AUC = 0.6061 for Logistic (logistic regression), AUC = 0.6423 for DT (decision tree), AUC = 0.6064 for ENet (elasticity network), AUC = 0.6349 for KNN (K-Nearest Neighbors), AUC = 0.6438 for LightGBM, AUC = 0.6601 for RF (random forest), AUC = 0.6439 for XGBoost (extreme gradient boosting), AUC = 0.6039 for SVM (support vector machine), and AUC = 0.6073 for MLP (multilayer perceptron).

### Cross-validation

**Fig 5:**
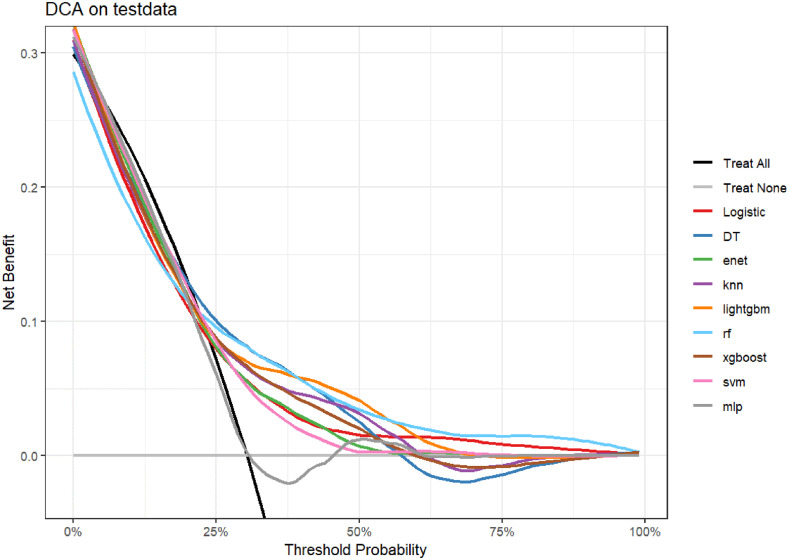
DCA curves for nine machine learning models.

**Fig 6:**
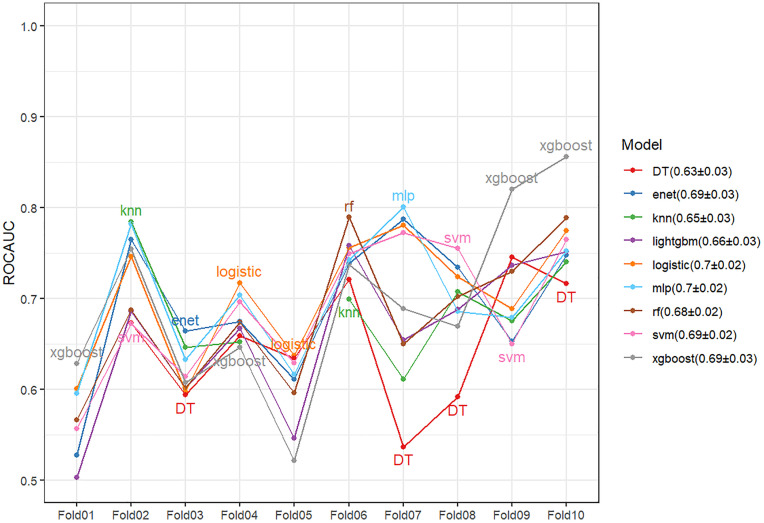
10-fold cross-validation of nine machine learning models.

The line chart of the cross-validated models (Fig 5) was obtained after 10 cross-validations, with the average ROC AUC ± standard deviation of each model shown in the Figs. Fig 6 shows 0.63 ± 0.03 for DT (Decision Tree), 0.69 ± 0.03 for ENet, 0.65 ± 0.02 for KNN (K-Nearest Neighbors), 0.66 ± 0.03 for LightGBM, 0.70 ± 0.02 for Logistic, 0.70 ± 0.02 for MLP (multilayer perceptron), 0.68 ± 0.02 for RF (random forest), 0.69 ± 0.02 for SVM (support vector machine), and 0.69 ± 0.03 for XGBoost.

### Cox analysis of 28-day survival for S/F

**Fig 7:**
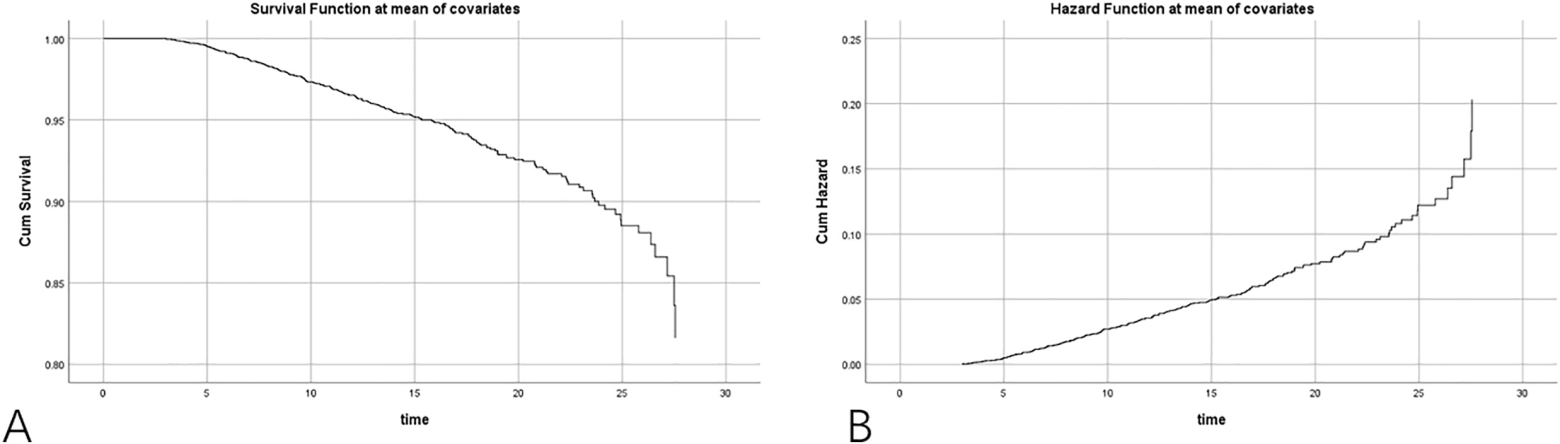
Cox regression of S/F for 28-day survival. A: survival function plot and B: hazard function plot.

Table 5 shows B (regression coefficient) of −0.095, SE (standard error) of 0.012, Wald (Wald statistic) of 62.821, DF (degrees of freedom) of 1, Sig. (significance level) of <0.001, Exp(B) (risk ratio) of 0.909, 95.0% CI for Exp(B) (95% Confidence Interval of risk ratio) of (0.888, 0.931), with the significance of S/F less than 0.01 indicating statistically significant. Fig 7A shows a gradual decrease in survival rate with increasing time. Fig 7B shows the risk of events gradually increases with time.

**Table 5:** Cox analysis of S/F for 28-day survival.

Variables in the Equation								
	B	SE	Wald	df	Sig.	Exp(B)	95.0% CI for Exp(B)	
							Lower	Upper
S/F7	−0.095	0.012	62.821	1.000	<0.001	0.909	0.888	0.931

### S/P*F ranges for different Berlin definitional grades

**Fig 8:**
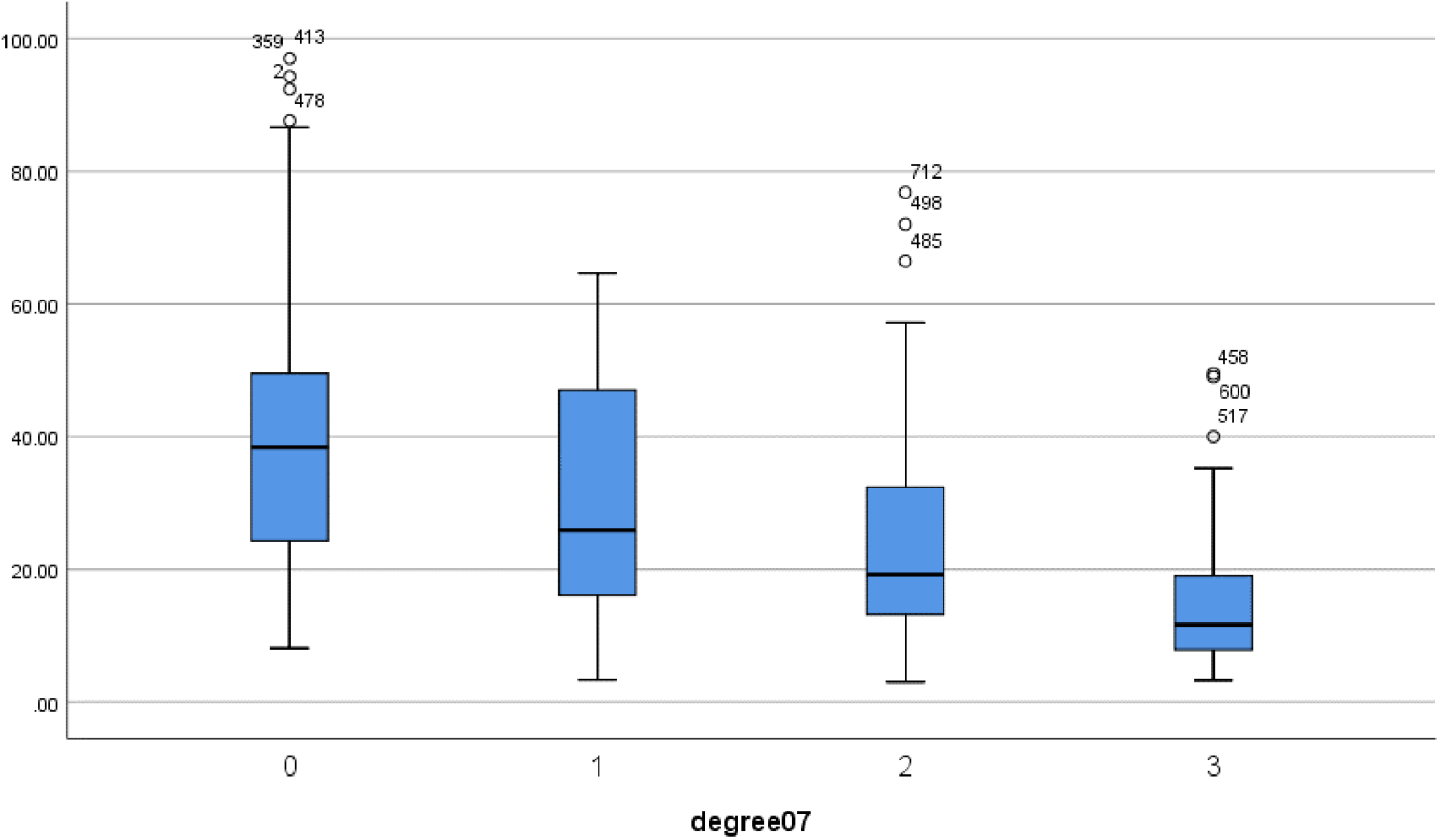
Box plot of the distribution of S/F*P values under different seventh day Berlin definition classifications.

Supplementary S1 Table in S1 File and Fig 8 show that Patients who are not ARDS have degree07 = 0, Mean of 39.067, 95% Confidence Interval of [35.881, 42.252], 5% Trimmed Mean of 38.0378, Median of 38.400. Patients with mild ARDS have degree07 = 1, and Mean of 29.476, 95% Confidence Interval of [27.387, 31.564], 5% Trimmed Mean of 29.064, and Median of 25.881. Patients with moderate ARDS have degree07 = 2, Mean of 23.556, 95% confidence interval of [22.184, 24.927], 5% Trimmed Mean of 22.875, and Median of 19.200. Patients with severe ARDS have degree07 = 3, Mean of 14.255, 95% Confidence interval of [12.747, 15.764], 5% Trimmed Mean of 13.445, and Median of 11.644. ROC analysis was carried out for mild-moderate and moderate-severe patients and yielded cutoff values of 24.460 and 12.700, respectively. Based on the S/F classification and the average value of the sought PEEP(Mean = 10), we propose that for mild ARDS patients, 23.5 < S/F * P ≤ 31.5; for moderate ARDS patients, 14.8 < S/F * P ≤ 23.5; and for severe ARDS patients, S/F * P ≤ 14.8.

## Discussion

It should be noted that all patients included in this study were clinically diagnosed with ARDS; therefore, our objective was to evaluate the relative diagnostic performance of oxygenation indices in classifying ARDS severity, rather than distinguishing ARDS from non-ARDS conditions. Within this homogeneous population, S/F*P showed slightly improved discrimination of severity compared with traditional indices such as S/F and P/F*P. However, when assessing prognostic prediction, the AUC values of all indices (0.55–0.70) indicated only poor-to-fair discrimination, suggesting that these formulas are more useful as auxiliary grading and monitoring tools than as independent predictors of mortality. Recently, it has been suggested that incorporating PEEP into the P/F ratio can reflect pulmonary compliance and lung recovery when assessing the severity of ARDS [7]. ROC curve calculations were performed by including PEEP in the newly proposed diagnostic criteria of SpO_2_/ FiO_2_ and PEEP in the P/F (P/F*P) for comparison. The results showed ROC_AUC = 0.700 (95 CI:0.624 ~ 0.777) for S/F, ROC_AUC = 0.720 (95 CI:0.668 ~ 0.772) for P/F*P, and ROC_AUC = 0.761(95 CI:0.693 ~ 0.930) for S/F*P, indicating that S/F had a better fit in diagnosing ARDS with slightly inferior efficacy to P/F*P, but the diagnostic efficacy was improved more significantly after incorporating PEEP into S/F, which further illustrated the validity of PEEP incorporation into S/F diagnosis.

The ability of S/F, S/F*P, P/F*P, and P/F to model patient prognosis was analyzed and a graded comparison was performed with PEEP≥8 because of the inclusion of PEEP, which ultimately resulted in S/F (S/F) of 0.598 and 0.603, S/F*P (S/FS/F*P) of 0.585 and 0.589, P/F*P (P/FP/F*P) of 0.608 and 0.600, and P/F (P/F) of 0.567 and 0.533 at day 3, as well as S/F (S/F) of 0.696 and 0.710, S/F*P (S/FS/F*P) of 0.686 and 0.698, P/F*P (P/FP/F*P) of 0.650 and 0.639, and P/F (P/F) of 0.626 and 0.633 at day 7, indicating that S/F and S/F*P demonstrated statistically significant but modest predictive ability for patient outcomes.The ROC AUC values ranging between 0.55 and 0.70 fall within the “poor-to-fair” range, suggesting that these indices should be interpreted as auxiliary markers for early evaluation rather than independent prognostic tools.Their clinical utility lies primarily in supporting timely oxygenation assessment and risk stratification rather than in accurately predicting mortality. It was found that the predictive ability of S/F*P for patients with ARDS still lags behind that of S/F but is still better than that of P/F*P and P/F at the same level despite the inclusion of PEEP in the calculations. With the increase in PEEP, the ability of the formulas in all groups except P/F*P tended to increase, which might be related to the fact that the increased P/F could not reflect the real situation of the patients after the PEEP increase, but it still needs to be verified by further studies. It was finally concluded that P/F had the best predictive ability, whose Youden’s index was calculated to be 181.90 at the maximum, which was used as the cutoff value, based on which the patient with S/F value lower or equal to 181.90 is considered to be at a higher risk of death. In clinical practice, this cutoff value can help physicians make more targeted treatment decisions. Closer monitoring and more aggressive therapeutic measures may be needed for those patients with S/F values below 181.90. This cutoff value can also be used for prognosis assessment, with patients with S/F values below this threshold requiring more attention and possible interventions to reduce the risk of death.

Random Forest (RF) achieved the highest score on ROC AUC (0.660), followed by XGBoost (0.644) and LightGBM (0.644). RF also had a relatively high PR AUC (0.536), indicating its good performance in actual classification ability. Balanced Accuracy reflects the model’s performance in handling imbalanced data: LightGBM had the highest balanced accuracy (0.642), followed by XGBoost (0.638) and Random Forest (0.606). Sensitivity and Specificity: LightGBM performed well in both sensitivity and specificity, effectively capturing both positive and negative categories. From the decision curve, Random Forest and LightGBM had higher net benefits at most thresholds. In the cross-validation plot, Random Forest showed more stable performance with less fluctuation. Based on the above analysis, Random Forest (RF) and LightGBM are the best choices. Due to Random Forest’s better stability, it is selected as the optimal model.

In Table 5, B (Coefficient): −0.095 is the regression coefficient of S/F7, which indicates that the log-odds of an event decrease by 0.095 for each unit increase in S/F7. Sig. (Significance): 0 represents the p-value, the very small of which (much less than 0.05) indicates that S/F7 has a significant effect on the model prediction. Exp(B) (Odds Ratio): 0.909 is the Odds Ratio, which indicates that the event rate decreases by 9.1% for each unit increase in S/F7. 95.0% CI for Exp(B) (95% Confidence Interval for Odds Ratio): 0.888 to 0.931 is the 95% confidence interval for the Odds Ratio excluding 1, which further supports the conclusion that S/F7 has a significant effect on the event rate. These results indicate that S/F is a significant predictor in the model, which means that an increase in S/F is associated with an increase in survival time under the control of other variables. Specifically speaking, the risk of an event is reduced by approximately 9.1% for each unit increase in S/F, with the effect being statistically significant.

**Fig 9:**
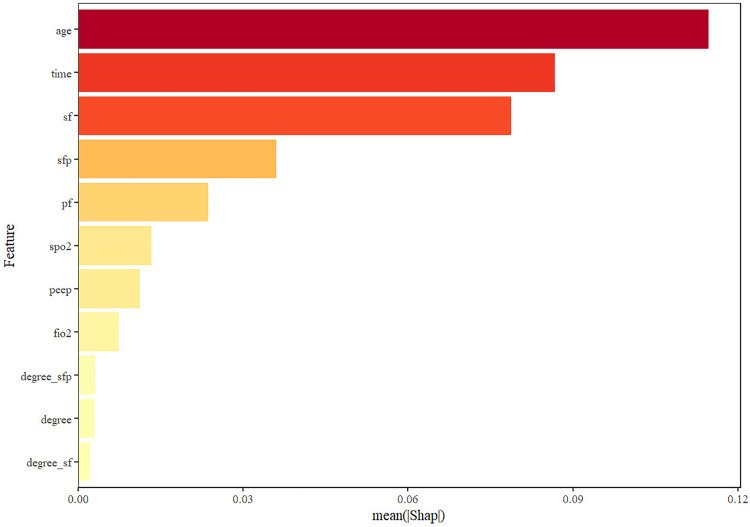
Importance distribution after lightGBM learning.

**Fig 10:**
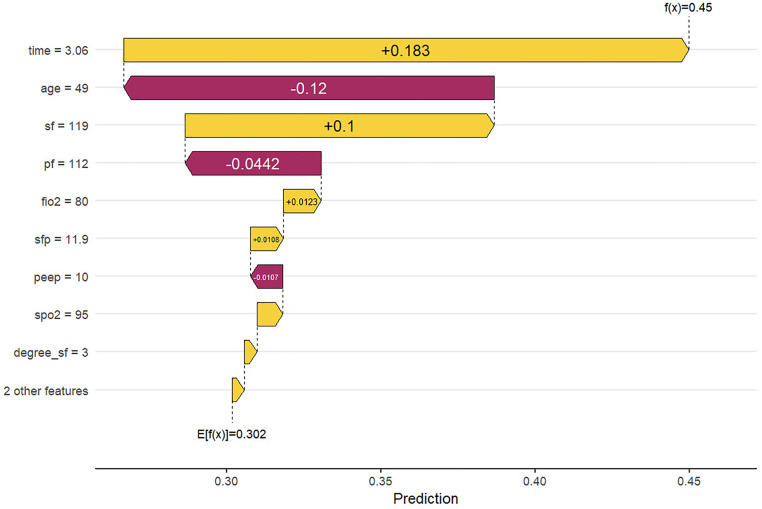
Feature values after lightGBM learning.

**Fig 11:**
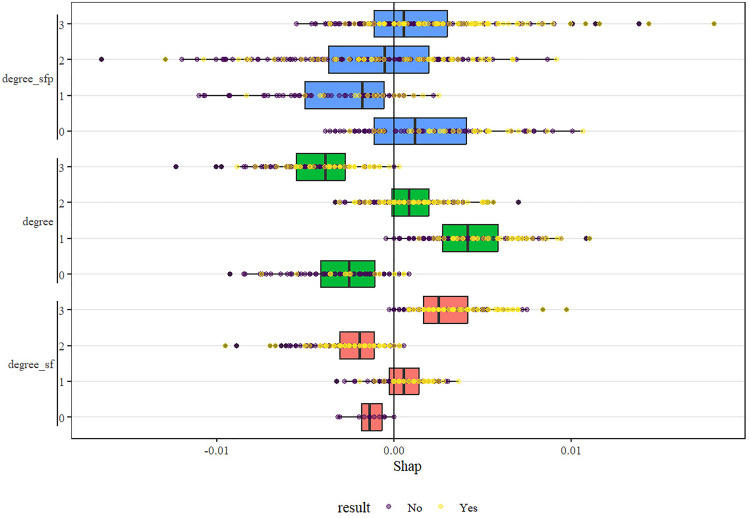
Distribution of SHAP values for different definition levels after lightGBM learning.

**Fig 12:**
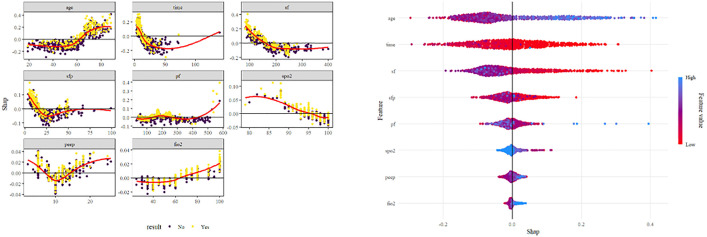
Distribution of SHAP values of different elements after lightGBM learning.

The performance of all machine learning models remained modest, with AUC values below 0.75 across algorithms. This finding aligns with the results of traditional ROC analysis, suggesting that oxygenation indices alone have limited discriminative power for predicting outcomes in ARDS. The use of machine learning in this study was intended for exploratory validation rather than clinical application. These results highlight that integrating additional physiologic and clinical variables—such as ventilator settings, comorbidities, and inflammatory markers—may be necessary to enhance predictive accuracy in future studies. Among all machine learning algorithms, the random forest model was selected for SHAP analysis because it exhibited relatively stable performance and provided better interpretability for feature contribution visualization (Figs 9–12). Analysis of the average absolute SHAP values (Feature Importance graph) revealed that degree_S/FS/F*P had the highest contribution, followed by degree_S/F, while degree had the least impact. The significant contributions of degree_S/FS/F*P and degree_S/F suggest that these features capture the complexity of patient conditions more effectively. In the SHAP value box plots, degree_S/FS/F*P showed a clearer distinction between positive and negative predictions, indicating its strong discriminatory power. Degree_S/F and degree exhibited weaker influences, particularly with degree’s distribution clustering around zero, which suggests limited discrimination capability for model predictions. Among the degree series, degree_S/F,S/F*P emerged as the most important feature, exerting the greatest influence and demonstrating robust predictive power. From the SHAP importance graph, the oxygenation index (S/F) had the highest average SHAP value, followed by S/F,S/F*P and P/F. This indicates that S/F is the most critical factor for model prediction, with S/F,S/F*P and P/F contributing progressively less. The wider distribution of S/F’s SHAP values highlights its substantial impact on both positive and negative classifications. In contrast, the narrower distributions of S/F,S/F*P and P/F suggest their relatively lower influence on model predictions. SHAP interaction analysis revealed that higher S/F values were generally associated with lower prediction risks, confirming S/F’s role as the most important indicator among S/F, S/F,S/F*P, and P/F. This underscores that S/F better reflects the physiological information relevant to prediction in this model.

It was mentioned in a study on the effect of PEEP on AECC definition by Elisa Estenssoro et al. that the diagnosis of patients with ARDS by PaO_2_/ FiO_2_ masks ARDS as hypoxemia improves with increasing PEEP [22]. The Berlin definition of PEEP only makes a provision for PEEP ≥5 mmH_2_O. Therefore, the question that improved oxygenation indices with the elevated PEEP because of the gradually elevated treatment requirements is caused by patient’s improved lung condition or greater exogenous PEEP remains unclear. It is not advisable to ignore lung damage because of elevated PaO_2_/ FiO_2_ [23]. The “lung opening” strategy proposed by B. Lachmann has become a common clinical strategy for protective ventilation, including lung recruitment maneuvers and maintenance of continuous alveolar stabilization. For example, PEEP can be gradually increased to 35 mmH_2_O in ventilatory PVC mode [24,25]. However, a high PEEP is still needed to maintain the lungs after opening, with drawbacks in the aspects of how the progression of ARDS patients during this period can be determined.

SpO_2_/ FiO_2_ can be used to diagnose ARDS in resource-limited areas, with its accuracy reduced in poor perfusion or oxygen saturation above 97%, as well as in patients with darker skin pigmentation. Therefore, the recognition ability of SpO_2_/ FiO_2_ needs to be determined by further prospective studies [26]. There is still a need for a better and simpler method for early recognition of ARDS. PEEP has been introduced to help in diagnosis, allowing real-time assessment of patient condition. Unfortunately, there was no significant improvement in predicting patient prognosis.

It was suggested in a study by Tommaso Mauri et al. that higher positive end-expiratory pressures and lower levels of support increased the proportion of tidal ventilation that reaches the dependent lung regions in patients with acute respiratory distress syndrome receiving pressure support ventilation, thus producing more homogeneous ventilation [27]. High airway pressures have been proposed to open collapsed ARDS lungs and partially open edematous lungs in a study by Valente Barbas et al. High PEEP levels and low tidal volume ventilation can reduce bronchoalveolar and plasma inflammatory mediators to improve survival compared to low PEEP/high tidal volume ventilation. It is needed to apply more sensitive information to determine the lung condition and adjust PEEP strategies on time when attempting to achieve better therapeutic outcomes by increasing PEEP.

There are still some limitations in the study. For example, the study was performed only based on the existing data in the database and lacked more precise and dynamic clinical data for reference. In the database, the confirmed severe patients occupy the majority, which may have a certain impact on the data.In addition, only the diagnostic formula for ARDS was investigated in the study, which still needs to be further improved despite its statistical significance in the prediction of patient prognosis. Therefore, further research is needed to investigate the assessment ability of S/F*P on the prognosis of ARDS patients in different settings.

## Conclusion

In the study, a new formula SpO_2_*10/(FiO_2_*PEEP) (S/F*P) with diagnostic and predictive value was provided by retrieving data from the MIMIC-IV v3.0 and eICU Collaborative Research Database v2.0databases in conjunction with the Berlin definition for ARDS patients. The stability and reliability of the model were further tested by nine machine learning and cross-validation methods, and a new corresponding grading of ARDS was proposed to assist the diagnosis by combining the statistical methods, which improved the ARDS diagnosis. In addition, the comparison results showed that this formula has a better diagnostic value than (PaO_2_ * 10)/(FiO_2_ * PEEP) (P/F*P), SpO_2_/ FiO_2_ (S/F), and other formulas. In terms of predicting the prognosis of patients, the ability of S/F*P is not as good as S/F, but the grading of S/F*P has a more positive significance for the evaluation of the prognosis of patients.The study also provides a more convenient way of judgment for mechanically ventilated patients, which can try to avoid the phenomenon of clinical diagnosis of PEEP separating from oxygenation index, as well as improve the choice of ARDS treatment method, providing a reference for the early treatment of ARDS in the clinic, helping to improve the resource allocation in the ICU and reduce the mortality rate of patients.

Given that all patients in this study had a confirmed diagnosis of ARDS, the analyses focused on the relative diagnostic performance of different oxygenation indices rather than on disease versus non-disease discrimination. Although additional physiological parameters—such as tidal volume, plateau pressure, mean airway pressure, and ventilation mode—are clinically relevant, these variables contained substantial missing data. Including them would have drastically reduced the effective sample size and compromised both statistical validity and external generalizability; thus, they were excluded from the final analysis.

While hemoglobin and sepsis were incorporated and the SOFA score was used to adjust for systemic dysfunction, other patient-related factors, particularly detailed comorbidities, were incompletely recorded. Incorporating these parameters would have markedly decreased the available sample size and weakened model robustness, which remains an inherent limitation of this retrospective database study.

Future prospective studies with standardized and comprehensive data collection are warranted to confirm and extend these findings. Moreover, the relatively low AUC values (mostly below 0.7) indicate that the prognostic capacity of these indices remains limited; therefore, they should be interpreted as auxiliary tools for early evaluation rather than as independent predictors of outcomes without further external validation.

## Supporting information

S1 File Descriptive statistics of S/F*P (SFP7) values across different Berlin definition grades on day 7. (DOCX)

## Abbreviations

ARDS: Acute Respiratory Distress Syndrome
AUC: Area Under the Receiver Operating Characteristic Curve
CI: Confidence Interval
CORR: Correlation of Predicted and Measured Values of Target Variables
ECMO: Extracorporeal Membrane Oxygenation
eICU Collaborative Research Database v2.0: eICU Collaborative Research Database v2.0
FiO₂: Fraction of Inspired Oxygen
SpO₂: Peripheral Oxygen Saturation
PaO₂: Partial Pressure of Arterial Oxygen
S/F: (SpO₂ * 10)/FiO₂
S/F*P: (SpO₂ * 10)/(FiO₂ * PEEP)
P/F*P: PaO₂/(FiO₂ * PEEP)
MLA: Machine learning algorithms
DT: Decision Tree
RF: Random Forest
ENet: Elastic Net
SVM: Support Vector Machine
MLP: Multi-Layer Perceptron
KNN: K-Nearest Neighbors
XGBoost: Extreme Gradient Boosting
LightGBM: Light Gradient Boosting Machine

## References

[1] Ware LB, Matthay MA. The acute respiratory distress syndrome. N Engl J Med. 2000;342(18):1334–49. doi: 10.1056/NEJM200005043421806 10793167

[2] Fanelli V, Ranieri VM. Mechanisms and clinical consequences of acute lung injury. Ann Am Thorac Soc. 2015;12 Suppl 1:S3-8. doi: 10.1513/AnnalsATS.201407-340MG 25830831

[3] ARDS Definition Task Force, Ranieri VM, Rubenfeld GD, Thompson BT, Ferguson ND, Caldwell E, et al. Acute respiratory distress syndrome: the Berlin definition. JAMA. 2012;307(23):2526–33. doi: 10.1001/jama.2012.5669 22797452

[4] Matthay MA, Arabi Y, Arroliga AC, Bernard G, Bersten AD, Brochard LJ, et al. A new global definition of acute respiratory distress syndrome. Am J Respir Crit Care Med. 2024;209(1):37–47. doi: 10.1164/rccm.202303-0558WS 37487152 PMC10870872

[5] Meyer NJ, Gattinoni L, Calfee CS. Acute respiratory distress syndrome. Lancet. 2021;398(10300):622–37. doi: 10.1016/S0140-6736(21)00439-6 34217425 PMC8248927

[6] Palanidurai S, Phua J, Chan YH, Mukhopadhyay A. P/FP ratio: incorporation of PEEP into the PaO2/FiO2 ratio for prognostication and classification of acute respiratory distress syndrome. Ann Intensive Care. 2021;11(1):124. doi: 10.1186/s13613-021-00908-3 34370116 PMC8350287

[7] Kamo T, Tasaka S, Suzuki T, Asakura T, Suzuki S, Yagi K, et al. Prognostic values of the Berlin definition criteria, blood lactate level, and fibroproliferative changes on high-resolution computed tomography in ARDS patients. BMC Pulm Med. 2019;19(1):37. doi: 10.1186/s12890-019-0803-0 30744598 PMC6371514

[8] Mélot C. Contribution of multiple inert gas elimination technique to pulmonary medicine. 5. Ventilation-perfusion relationships in acute respiratory failure. Thorax. 1994;49(12):1251–8. doi: 10.1136/thx.49.12.1251 7878564 PMC475336

[9] Ball L, Serpa Neto A, Trifiletti V, Mandelli M, Firpo I, Robba C, et al. Effects of higher PEEP and recruitment manoeuvres on mortality in patients with ARDS: a systematic review, meta-analysis, meta-regression and trial sequential analysis of randomized controlled trials. Intensive Care Med Exp. 2020;8(Suppl 1):39. doi: 10.1186/s40635-020-00322-2 33336325 PMC7746429

[10] Alhurani RE, Oeckler RA, Franco PM, Jenkins SM, Gajic O, Pannu SR. Refractory Hypoxemia and use of rescue strategies. A U.S. national survey of adult intensivists. Ann Am Thorac Soc. 2016;13(7):1105–14. doi: 10.1513/AnnalsATS.201508-560OC 27128143

[11] Edginton S, Kruger N, Stelfox HT, Brochard L, Zuege DJ, Gaudet J, et al. Methods for determining optimal positive end-expiratory pressure in patients undergoing invasive mechanical ventilation: a scoping review. Can J Anaesth. 2024;71(11):1535–55. doi: 10.1007/s12630-024-02871-6 39565498 PMC11602853

[12] Dushianthan A, Cusack R, Chee N, Dunn J-O, Grocott MPW. Perceptions of diagnosis and management of patients with acute respiratory distress syndrome: a survey of United Kingdom intensive care physicians. BMC Anesthesiol. 2014;14:87. doi: 10.1186/1471-2253-14-87 25309125 PMC4192350

[13] Slutsky AS, Ranieri VM. Ventilator-induced lung injury. N Engl J Med. 2014;370(10):980. doi: 10.1056/NEJMc1400293 24597883

[14] Todur P, Nileshwar A, Chaudhuri S, Gupta N, Natarajan S, Rao S. Utility of Pulse Oximetry Oxygen Saturation (SpO2) with Incorporation of Positive End-Expiratory Pressure (SpO2*10/FiO2*PEEP) for classification and prognostication of patients with acute respiratory distress syndrome. Crit Care Res Pract. 2022;2022:7871579. doi: 10.1155/2022/7871579 36111248 PMC9470362

[15] Johnson AEW, Bulgarelli L, Shen L, Gayles A, Shammout A, Horng S, et al. Author Correction: MIMIC-IV, a freely accessible electronic health record dataset. Sci Data. 2023;10(1):219. doi: 10.1038/s41597-023-02136-9 37072428 PMC10113185

[16] Goldberger AL, Amaral LA, Glass L, Hausdorff JM, Ivanov PC, Mark RG, et al. PhysioBank, PhysioToolkit, and PhysioNet: components of a new research resource for complex physiologic signals. Circulation. 2000;101(23):E215-20. doi: 10.1161/01.cir.101.23.e215 10851218

[17] Pollard T, Johnson A Raffa J, Celi L, Badawi O, Mark R. eICU collaborative research database. PhysioNet. 2019. doi: 10.13026/C2WM1R PMC6132188 30204154

[18] Pollard TJ, Johnson AEW, Raffa JD, Celi LA, Mark RG, Badawi O. The eICU Collaborative Research Database, a freely available multi-center database for critical care research. Sci Data. 2018;5:180178. doi: 10.1038/sdata.2018.178 30204154 PMC6132188

[19] Sayed M, Riaño D, Villar J. Novel criteria to classify ARDS severity using a machine learning approach. Crit Care. 2021;25(1):150. doi: 10.1186/s13054-021-03566-w 33879214 PMC8056190

[20] Hou F, Zhu Y, Zhao H, Cai H, Wang Y, Peng X, et al. Development and validation of an interpretable machine learning model for predicting the risk of distant metastasis in papillary thyroid cancer: a multicenter study. EClinicalMedicine. 2024;77:102913. doi: 10.1016/j.eclinm.2024.102913 39552714 PMC11567106

[21] The Lancet Respiratory Medicine. Opening the black box of machine learning. Lancet Respir Med. 2018;6(11):801. doi: 10.1016/S2213-2600(18)30425-9 30343029

[22] Lachmann B. Open up the lung and keep the lung open. Intensive Care Med. 1992;18(6):319–21. doi: 10.1007/BF01694358 1469157

[23] Zhang H, Qian D, Zhang X, Meng P, Huang W, Gu T, et al. Tree-based ensemble machine learning models in the prediction of acute respiratory distress syndrome following cardiac surgery: a multicenter cohort study. J Transl Med. 2024;22(1):772. doi: 10.1186/s12967-024-05395-1 39148090 PMC11325832

[24] Erratum: An official american thoracic society/european society of intensive care medicine/society of critical care medicine clinical practice guideline: mechanical ventilation in adult patients with acute respiratory distress syndrome. Am J Respir Crit Care Med. 2017;195(11):1540. doi: 10.1164/rccm.19511erratum 28569586

[25] Wick KD, Matthay MA, Ware LB. Pulse oximetry for the diagnosis and management of acute respiratory distress syndrome. Lancet Respir Med. 2022;10(11):1086–98. doi: 10.1016/S2213-2600(22)00058-3 36049490 PMC9423770

[26] Mauri T, Bellani G, Confalonieri A, Tagliabue P, Turella M, Coppadoro A, et al. Topographic distribution of tidal ventilation in acute respiratory distress syndrome: effects of positive end-expiratory pressure and pressure support. Crit Care Med. 2013. 10.1097/CCM.0b013e318287f6e7 23507723

[27] Chiumello D, Cressoni M, Carlesso E, Caspani ML, Marino A, Gallazzi E, et al. Bedside selection of positive end-expiratory pressure in mild, moderate, and severe acute respiratory distress syndrome. Crit Care Med. 2014;42(2):252–64. doi: 10.1097/CCM.0b013e3182a6384f 24196193

