# Supplementary material for: Evaluation of oxygenation indices incorporating SpO₂ and PEEP for assessing ARDS severity: Evidence from the MIMIC-IV and eICU collaborative research database v2.0 databases
Source: PLoS One. 2026 Feb 5;21(2):e0341004. doi: 10.1371/journal.pone.0341004 (PMC12875482; doi:10.1371/journal.pone.0341004)
Supplement: S1 File — (DOCX) [file pone.0341004.s001.docx]

| Descriptives | | | | | |
| --- | --- | --- | --- | --- | --- |
| **degree07** | | | | **Statistic** | **Std. Error** |
| **SFP7** | **0** | **Mean** | | **39.0667** | **1.61095** |
|  |  | **95% Confidence Interval for Mean** | **Lower Bound** | **35.8814** |  |
|  |  |  | **Upper Bound** | **42.2521** |  |
|  |  | **5% Trimmed Mean** | | **38.0378** |  |
|  |  | **Median** | | **38.4000** |  |
|  |  | **Variance** | | **360.727** |  |
|  |  | **Std. Deviation** | | **18.99282** |  |
|  |  | **Minimum** | | **8.08** |  |
|  |  | **Maximum** | | **97.00** |  |
|  |  | **Range** | | **88.92** |  |
|  |  | **Interquartile Range** | | **25.50** |  |
|  |  | **Skewness** | | **0.687** | **0.206** |
|  |  | **Kurtosis** | | **0.386** | **0.408** |
|  | **1** | **Mean** | | **29.4755** | **1.05921** |
|  |  | **95% Confidence Interval for Mean** | **Lower Bound** | **27.3868** |  |
|  |  |  | **Upper Bound** | **31.5642** |  |
|  |  | **5% Trimmed Mean** | | **29.0640** |  |
|  |  | **Median** | | **25.8810** |  |
|  |  | **Variance** | | **224.385** |  |
|  |  | **Std. Deviation** | | **14.97950** |  |
|  |  | **Minimum** | | **3.30** |  |
|  |  | **Maximum** | | **64.67** |  |
|  |  | **Range** | | **61.37** |  |
|  |  | **Interquartile Range** | | **30.96** |  |
|  |  | **Skewness** | | **0.424** | **0.172** |
|  |  | **Kurtosis** | | **-1.091** | **0.342** |
|  | **2** | **Mean** | | **23.5555** | **0.69768** |
|  |  | **95% Confidence Interval for Mean** | **Lower Bound** | **22.1836** |  |
|  |  |  | **Upper Bound** | **24.9274** |  |
|  |  | **5% Trimmed Mean** | | **22.8746** |  |
|  |  | **Median** | | **19.2000** |  |
|  |  | **Variance** | | **180.098** |  |
|  |  | **Std. Deviation** | | **13.42005** |  |
|  |  | **Minimum** | | **3.00** |  |
|  |  | **Maximum** | | **76.80** |  |
|  |  | **Range** | | **73.80** |  |
|  |  | **Interquartile Range** | | **19.22** |  |
|  |  | **Skewness** | | **0.906** | **0.127** |
|  |  | **Kurtosis** | | **0.272** | **0.253** |
|  | **3** | **Mean** | | **14.2554** | **0.76264** |
|  |  | **95% Confidence Interval for Mean** | **Lower Bound** | **12.7469** |  |
|  |  |  | **Upper Bound** | **15.7639** |  |
|  |  | **5% Trimmed Mean** | | **13.4450** |  |
|  |  | **Median** | | **11.6442** |  |
|  |  | **Variance** | | **77.938** |  |
|  |  | **Std. Deviation** | | **8.82824** |  |
|  |  | **Minimum** | | **3.21** |  |
|  |  | **Maximum** | | **49.50** |  |
|  |  | **Range** | | **46.29** |  |
|  |  | **Interquartile Range** | | **11.17** |  |
|  |  | **Skewness** | | **1.498** | **0.209** |
|  |  | **Kurtosis** | | **2.829** | **0.416** |
